# Gastric Antral Gastrointestinal Stromal Tumor Presenting With Severe Anemia

**DOI:** 10.7759/cureus.32728

**Published:** 2022-12-20

**Authors:** Wendolin J Ortiz, Samanta Landazuri-Navas, Nora Moron-Cabrera, Alberto Calle-Encalada, Geovanny Gutierrez-Brito, Esmeralda Vilchez, Gail Fernandes, Evelyn Calderon-Martinez

**Affiliations:** 1 General Surgery, Universidad Autónoma de Baja California, Mexicali, MEX; 2 Internal Medicine, Universidad de las Américas, Quito, ECU; 3 Family Medicine, Mayagüez Medical Center, Mayagüez, PRI; 4 Internal Medicine, Universidad de Guayaquil, Guayaquil, ECU; 5 Internal Medicine, Universidad Autónoma de Guadalajara, Guadalajara, MEX; 6 Internal Medicine, Baylor College of Medicine, Houston, USA; 7 Internal Medicine, University of Pittsburgh Medical Center, Harrisburg, USA

**Keywords:** gist, tumor imaging, gastrointestinal bleeding, anemia, gastrointestinal stromal tumor

## Abstract

Gastrointestinal stromal tumors are rare gastrointestinal tract growths associated with high rates of malignant transformation. Most cases are asymptomatic and can be identified by computed tomography scan. We present the case of a 50-year-old male with melena and fatigue. Endoscopy showed an ulcerated submucosal tumor diagnosed as a gastrointestinal stromal tumor after surgical resection; it did not present with metastasis and was successfully treated surgically without relapse.

## Introduction

Gastrointestinal stromal tumors (GISTs) are rare gastrointestinal tract growths originating from the interstitial cells of Cajal. Most cases are asymptomatic and identified incidentally by endoscopy or computed tomography (CT) scan. They have great potential for malignant transformation and account for 1%-2% of gastrointestinal neoplasms; unfortunately, chemotherapy and radiation are ineffective, so surgical resection has always been the mainstream treatment. As the knowledge regarding mutations associated with these tumors increases, therapy has evolved. The selective tyrosine kinase receptors inhibitor (TKI), imatinib mesylate, is used as an adjuvant or neoadjuvant therapy that improves the risk of local recurrence and metastasis. However, due to tumor resistance, sunitinib and regorafenib are effective second-line TKIs [1].

## Case presentation

Our patient is a 50-year-old man who presented to the outpatient clinic complaining of a one-month history of black stools, nausea, fatigue, and dyspnea on exertion. His past medical history was remarkable for hemorrhoids, and he had never had a colonoscopy or upper endoscopy. Upon interrogation, he denied abdominal pain. His family history was negative for stomach or colon cancer. He had no record of smoking, alcohol, or drug abuse.

**Figure 1:**
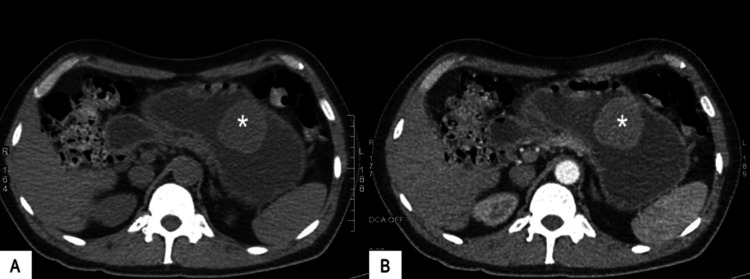
CT scan of the abdomen in the axial plane (A) CT scan of the abdomen without contrast revealing stomach-thickened walls and a large intragastric mass (asterisk) and (B) contrast-enhanced CT (arterial phase) showing the same lesion (asterisk). CT: Computed tomography.

**Figure 2:**
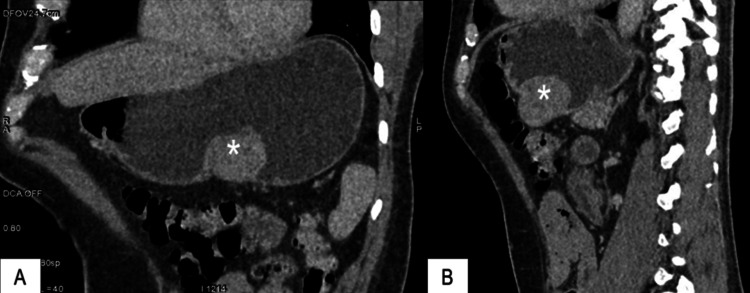
CT scan of the abdomen showing the lesion in coronal and sagittal planes (A) Coronal oblique reconstruction CT and (B) oblique sagittal reconstruction CT. CT: Computed tomography.

On physical examination, the patient had a severely pale appearance; the abdomen was soft and non-tender, and bowel sounds were normal. Rectal examination revealed an empty ampulla with fecal residues. His initial hemoglobin was 5.2 g/dL, and he received four doses of intravenous iron supplementation. Repeat blood work showed hemoglobin levels of 4.5 g/dL, which prompted the medical team to perform an endoscopy that showed an ulcerated submucosal tumor (IIb Forrest). A CT scan of the abdomen reported stomach-thickened walls of approximately 33 mm at the antrum level and a large pylorus (Figures 1, 2). The patient underwent a transfusion of four units of pRBC, and the mass resection was scheduled.

After the gastric tumor was laparoscopically resected and sent for histopathological evaluation, pathology determined a GIST measuring 5 cm x 10 cm, with a mitotic index of 5 mitosis/high power fields (HPF). Of note, the patient did not receive imatinib, and his six-month follow-up CT scan of the abdomen showed no signs of a remaining mass or metastasis.

## Discussion

Our patient with a history of hemorrhoids presented with melena, fatigue, nausea, dyspnea on exertion, and hemoglobin of 5.2 g/dL. Even though rare, bleeding internal hemorrhoids can lead to iron deficiency anemia and necessitate transfusion. The patient received four doses of intravenous iron supplementation. The repeated hemoglobin levels were 4.5 g/dL, and a subsequent endoscopy showed an ulcerated submucosal tumor (IIb Forrest) as the probable cause of his anemia.

Submucosal lesions are growths in the gastrointestinal tract covered with normal mucosa and may also be ulcerated or inflamed. Gastric submucosal tumors are usually incidentally found during routine health examinations without any symptoms, but sometimes, abdominal pain, palpable abdominal mass, and weight loss may present. The most prevalent are GIST, leiomyoma, neuroendocrine tumors, lipomas, granular cell tumors, heterotopic pancreas, Brunner's gland hamartoma, and lymphangiomas. By the site of origin, leiomyomas most frequently appear in the esophagus, lipomas in the right colon, and granular cell tumors in the lower and middle esophagus. Leiomyomas are pale, firm, rubbery masses characterized by a bland spindle cell population arranged in fascicles and whorls, with few mitoses, and immunohistochemistry (IHC) that is negative for CD34 and CD117. Schwannomas are benign gray masses characterized by spindle cells, lymphocytes, and a nodular lymphoid cuff, and they are positive for S100 and vimentin on IHC.Granular cell tumors are benign neurogenic tumors that appear as sheets of uniform histiocyte-like cells with abundant, eosinophilic, periodic acid-Schiff-reaction-positive cytoplasm containing lysosomal granules and small vesicular nuclei, and they are positive for S100 and neuron-specific enolase [2-5].

Our patient's final diagnosis was GISTs, which are neoplasms that are found along the gastrointestinal tract and located in the stomach more than 50% of the time. These tumors can go from a few millimeters to more than 20 cm in diameter, with a typical average of 5-8 cm; they are gray and well circumscribed but not encapsulated and may show signs of hemorrhage, calcification, ulceration, necrosis [1,3,6,7].

GISTs originate from the known peacemaker's gastrointestinal tract cells, the Cajal cells, which express two protein tyrosine kinase receptors in the muscularis propria: KIT and PDGFRA. A mutation in the c-kit gene causes the activation and gain of function of the enzymatic activity of the KIT type II transmembrane tyrosine kinase receptor affecting the intracellular signal transductions, which regulates proliferation, differentiation, and anti-apoptotic signaling. This causes a ligand-independent tyrosine kinase activity, resulting in uncontrolled cell proliferation and altered cell growth. The mutation in the PDGFRA receptor causes similar effects. Most GISTs are sporadic (97%), while the remaining percentage includes familial GIST syndromes such as neurofibromatosis and Carney-Stratakis syndrome [8].

GISTs represent about 1% of all primary malignant tumors and are the most common mesenchymal neoplasms of the gastrointestinal tract. The worldwide annual incidence in 2021 is approximately seven to 15 cases per million people, with 600 new patients diagnosed annually in the United States. The median age of the diagnosis is between 50 and 70 years old, with no predilection for either gender or known predisposing risk factors [1,3].

GISTs are usually asymptomatic until they reach a size of about 6 cm. These large tumors are often vascular and can present with pain or GI bleeding toward the gastrointestinal lumen or abdominal cavity. Less common presentations include nausea, pleuritic chest pain, pelvic pain, and small bowel obstruction. Approximately 20% of patients have metastasis when they are diagnosed. The most common sites of metastasis are the liver, abdominal cavity, and lymph nodes [9].

An echoendoscopy-guided biopsy is the technique of choice for pathological diagnosis, with CT-guided percutaneous biopsy as the second choice. Three histology types, namely fusiform, epithelioid, and mixed morphology, are described. CD117 protein is the most specific marker for GISTs on IHC [10].

The treatment of choice for localized GISTs is complete surgical resection with negative margins. GISTs rarely involve the regional lymph nodes, so extensive lymph node exploration or wide lymphadenectomy is rarely indicated. Small tumors/gastric GISTs (<5 cm) are managed laparoscopically, and minimally invasive techniques are submucosal tunneling endoscopic resection, endoscopic full-thickness resection, and laparoscopic, cooperative endoscopic surgery [1,3,11-13]. Open surgery is beneficial in the case of a large GIST to avoid rupture and decrease peritoneal dissemination. Neoadjuvant administration of imatinib, a TKI with potent activity against KIT, for three to six months can effectively diminish the size of tumors, thereby facilitating effective surgical intervention. Tumor resistance to this agent has been reported; this should be managed by either dose escalation or transition to treatment with sunitinib, a TKI with an antiangiogenic and antiproliferative activity that targets multiple kinases, including the vascular endothelial growth factor receptors, PDGFRA, KIT, and FLT3 [1,11,12,14].

Tumor size less than 5 cm in diameter, a complete surgical resection without tumor rupture, and low histologic grade of tumor are significant favorable prognostic factors. The recurrence rate following resection is 35% on average, and it has been reported that only 10% of these patients are disease-free on long-term follow-ups [15].

Seven cases of GIST presenting with moderate and mild anemia have been reported [16-22] in pediatric and adult patients. The clinical presentation included melena, fatigue, weight loss, attacks of vomiting, abdominal pain, and dyspnea. Hemoglobin in those patients ranged between 10.2 and 8.0 g/dL. Similar to our patient, one more case presented with severe anemia and hemoglobin of 5.5 g/dL but with multiple GISTs in the stomach and small intestine [20]. Our patient’s hemoglobin dropped to 0.7 g/dL after four doses of intravenous iron supplementation; three other cases mentioned that the hemoglobin tests also demonstrated a significant drop averaging 3 g/dL in two days, three months, and eight months despite the pharmacological treatment [18,21,22]. A prompt investigation of moderate anemia in a patient, considering GIST as a differential diagnosis, should be encouraged to avoid worsening hemoglobin levels and symptomatology.

## Conclusions

Patients with GIST can present with progressive anemia. Some concomitant diseases like hemorrhoids can be attributed as the cause of anemia, and GIST or other tumors can be missed. Other gastrointestinal disorders can be ruled out with the help of upper and lower endoscopies and a CT scan of the abdomen and pelvis. Patients with an ulcerated subepithelial mass, such as a GIST, causing bleeding should undergo surgical resection of the tumor to prevent rebleeding. Most GIST patients benefit from targeted molecular therapies. Even though they can be successfully treated with surgery, follow-up is mandatory due to the risk of recurrence.
